# Ideal Cardiovascular Health and Risk of Cardiovascular Events or Mortality: A Systematic Review and Meta-Analysis of Prospective Studies

**DOI:** 10.3390/jcm12134417

**Published:** 2023-06-30

**Authors:** Milan Radovanovic, Janko Jankovic, Stefan Mandic-Rajcevic, Igor Dumic, Richard D. Hanna, Charles W. Nordstrom

**Affiliations:** 1 Mayo Clinic College of Medicine and Science, Rochester, MN 55905, USA; 2 Department of Hospital Medicine, Mayo Clinic Health System, Eau Claire, WI 54703, USA; 3 Institute of Social Medicine, Faculty of Medicine, University of Belgrade, 11000 Belgrade, Serbia; 4 Centre-School of Public Health and Health Management, Faculty of Medicine, University of Belgrade, 11000 Belgrade, Serbia; 5 Department of Cardiology, Mayo Clinic Health System, Eau Claire, WI 54703, USA

**Keywords:** cardiovascular health, Life’s Simple 7, cardiovascular diseases and mortality

## Abstract

Cardiovascular diseases (CVD) remain the leading cause of morbidity and mortality worldwide, hence significant efforts have been made to establish behavior and risk factors associated with CVD. The American Heart Association proposed a 7-metric tool to promote ideal cardiovascular health (CVH). Recent data demonstrated that a higher number of ideal CVH metrics was associated with a lower risk of CVD, stroke, and mortality. Our study aimed to perform a systematic review and meta-analysis of prospective studies investigating the association of ideal CVH metrics and CVD, stroke, and cardiovascular mortality (CVM) in the general population. Medline and Scopus databases were searched from January 2010 to June 2022 for prospective studies reporting CVH metrics and outcomes on composite-CVD, coronary heart disease, myocardial infarction, stroke, and CVM. Each CVH metrics group was compared to another. Twenty-two studies totaling 3,240,660 adults (57.8% men) were analyzed. The follow-up duration was 12.0 ± 7.2 years. Our analysis confirmed that a higher number of ideal CVH metrics led to lower risk for CVD and CVM (statistically significant for composite-CVD, stroke, and CVM; *p* < 0.05). Conclusion: Even modest improvements in CVH are associated with CV-morbidity and mortality benefits, providing a strong public health message about the importance of a healthier lifestyle.

## 1. Introduction

Cardiovascular diseases (CVD) are major, global, non-communicable chronic diseases that are still the leading cause of morbidity and mortality within the United States (US) and worldwide despite declining age-standardized CVD-death rates over the second half of the 20th century [1,2]. The burden of CVD in terms of diminished quality of life, life-years lost, and direct and indirect medical costs remains substantial [2]. Nearly 50% of adults in the US have some form of CVD, and that number increases to nearly 60% among African Americans [3]. With life expectancy increasing over the past century, significant efforts have been made to establish health-related behaviors and health factors associated with CVD [1]. There is compelling evidence that unhealthy behaviors (e.g., smoking or a sedentary lifestyle) lead to unhealthy risk factors that worsen CVH and increase cardiovascular morbidity and mortality. This in turn leads to increased healthcare costs and financial burdens on individual, societal, and international levels [2]. Therefore, in 2010, the Goals and Metrics Committee of the Strategic Planning Task Force of the American Heart Association (AHA) proposed a seven-item tool as a part of their “2020 Impact Goals” to reduce the burden of CVD by promoting ideal CVH and primordial prevention [2,4]. The initial goal set in 2010 targeted a 20% reduction of death from CVD and stroke in the US via a 20% improvement of CVH in the American population [2]. This seven-item tool, also known as “Life’s Simple 7” (LS7), consists of four health-related behaviors (not smoking, healthy dietary intake, physical activity, and body mass index [BMI]), and three health factors (total cholesterol, blood pressure, and fasting plasma glucose) [2,4]. Each of the seven CVH metrics is classified further as either poor, intermediate, or ideal; in order to numerically categorize CVH, researchers have represented these metrics as numeric scores from 0 to 2 [2]. The AHA criteria for the definition of poor, intermediate, and ideal CVH metrics are presented in Supplement Table S1.

Recent data have demonstrated that the presence of a greater number of ideal CVH metrics was associated with a lower risk of CVD, stroke, and cardiovascular mortality (CVM) [5,6,7]. Since the inception of CVH, there have been numerous epidemiological studies on this topic (both cohort and cross-sectional); however, there have been very few systematic reviews and meta-analyses [5,6,7,8], with the latest being published in 2018 [7]. These earlier analyses had significant shortcomings in their design, methodology, and data interpretation, and were further limited by omitting some of the important studies [9].

## 2. The Aim of the Study

Our study aimed to perform a systematic review and meta-analysis of prospective cohort studies investigating the association of ideal CVH metrics and CVD (composite CVD, coronary heart disease [CHD], and MI), stroke, and CVM in the general population.

## 3. Materials and Methods

### 3.1. Search Strategy, Study Selection, and Quality Assessment

A comprehensive and systematic literature search of the Medline database (via the PubMed search engine) and the Scopus database was performed according to the preferred reporting items for systematic reviews and meta-analyses (PRISMA) guidelines from the inception of the CVH concept (2010) to 30 June 2022. The review was not registered. The following search keywords (a combination of MeSH and non-MeSH terms) were used: “ideal cardiovascular health”, “cardiovascular health metrics”, “Life’s Simple 7”, “cardiovascular diseases”, “coronary heart disease”, “stroke”, “cerebrovascular disease”, “mortality”, and “death”. Furthermore, the reference list of identified studies was manually screened to identify additional studies that can be included in our analysis.

Two authors (M.R. and I.D.) independently and blindly screened the titles, abstracts, and full manuscripts of the identified articles, excluding duplicates and articles irrelevant to the topic. Any discrepancies or uncertainties were resolved by a third author (J.J.).

Articles included in the study were eligible if they met the following criteria: written in English, peer-reviewed, observational prospective cohort studies investigating the ideal CVH metrics and reporting cardiovascular events (e.g., composite CVD, CHD, MI, or stroke) or CVM in the general adult population. Composite CVD represents a major CVD that was not specified in the studies. All eligible studies had reported adjusted relative risks (RR) or hazard ratios (HR) with confidence intervals (CI) or standard errors (SE). Authors of eligible studies with incomplete information were contacted to provide additional data; however, if this proved either impossible or ineffective, the study was rejected. Review articles, meta-analyses, commentaries and discussions, editorials, letters to editors (except when all relevant data was available), conference papers, books, or book chapters, as well as studies conducted on children, were excluded.

The Newcastle-Ottawa Scale (NOS) for cohort studies was used for methodological quality assessment. The NOS scale is a nine-star point system used to assess the quality of non-randomized studies, including cohort studies. The scale awards up to three stars in each of three categories: the selection of study groups; the comparability of the groups; and the ascertainment of the outcome of interest [10,11]. In our analysis authors M.R. and J.J. independently assessed the quality and calculated the NOS score for each study. Only high-quality studies with a NOS score of at least 7 were included in our analysis (Table 1).

### 3.2. Data Collection and Group Comparison

In addition to ideal CVH metrics, we extracted authors’ names, publication year, country of the study, study name, sample size, percentage of males, population age (average or range), number of cardiovascular events including CVM, and duration of follow-up. Based on the number of ideal CVH metrics, patients were categorized into 3 groups: poor CVH group (with the fewest ideal CVH metrics: between 0 and 2), intermediate CVH group (with CVH metrics between 3 and 4), and ideal CVH group (with CVH metrics between 5 and 7). In the studies where each of the seven CVH metrics was scored from 0 to 2, patients were categorized into the poor CVH group (score 0 to 4), intermediate CVH group (score between 5 and 9), and ideal CVH group (score 10 to 14). Data were presented as mean ± SD and median (interquartile range) for continuous variables or numbers (percentages) for categorical variables.

We compared the ideal CVH group (CVH metrics 5–7 or score 10–14) to the intermediate CVH group (CVH metrics 3–4 or score 5–9) and poor CVH group (CVH metrics 0–2 or score 0–4), as well as intermediate CVH group (CVH metrics 3–4 or score 5–9) to the poor CVH group (CVH metrics 0–2 or score 0–4).

### 3.3. Statistical Analysis

The analysis was carried out using the log risk ratio (RR) with 95% CI as the outcome measure comparing each CVH metrics group to another. The amount of heterogeneity (i.e., *Ʈ*^2^), was estimated using the restricted maximum-likelihood estimator [12]. In addition to the estimate of *Ʈ*^2^, the Cochrane Q-test for heterogeneity [13] and the *I*^2^ statistic [14] were reported. In case the *I*^2^ statistic was higher than 50%, a random-effects (RE) model was fitted to the data, otherwise, a fixed-effects (FE) model was fitted. Sensitivity analysis was performed to investigate the robustness of the findings and results, and to determine whether a particular study accounted for the heterogeneity. Studentized residuals and Cook’s distances were used to examine whether studies may be outliers and/or influential in the context of the model [15]. Studies with a studentized residual larger than the $100\times \left(1-0.05/\left(2\times k\right)\right)$th percentile of a standard normal distribution were considered potential outliers (i.e., using a Bonferroni correction with two-sided α=0.05 for k studies included in the meta-analysis). Studies with a Cook’s distance larger than the median plus 6 times the interquartile range of the Cook’s distances were considered influential. The presence of publication bias was assessed graphically by funnel plots. The rank correlation test [16] and the regression test (Egger) [17], using the SE of the observed outcomes as a predictor, were used to check for funnel plot asymmetry. The analysis was carried out using R Programming Language and Environment for Statistical Computing (version 4.2.2) [18] and the metafor package (version 3.8.1) [19]. Statistical significance was reported using a two-sided *p*-value of <0.05.

## 4. Results

### 4.1. Literature Search and Study Characteristics

**Figure 1:**
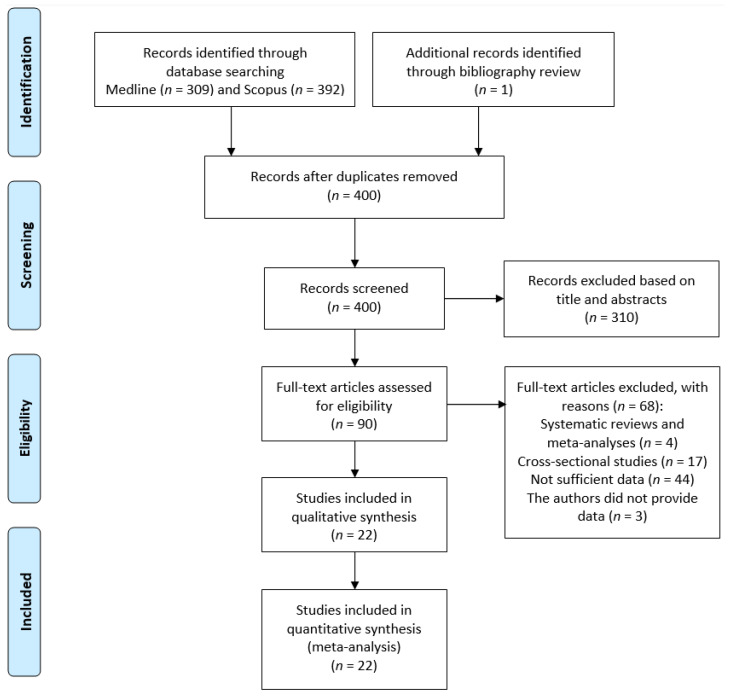
PRISMA flowchart detailing the search results.

The initial search of two databases (Medline and Scopus) over the span of 12 years yielded 701 records. One study that fulfilled inclusion criteria was manually identified by checking the reference lists of identified articles [20], totaling the number of analyzed records to 702. We screened the titles and abstracts of all 400 non-duplicate records and excluded 310 irrelevant articles for the topic. A total of 90 full-text articles were reviewed for eligibility, yielding 22 studies (articles) that met the eligibility criteria for our analysis. The flow chart of detailed article selection and the final studies included in the analysis was created (Figure 1). Three studies [21,22,23] did not have sufficient data on CVH metric groups, however, we were able to receive additional data from the authors of one study, which we included in our analysis [21].

All 22 selected and analyzed studies were observational prospective cohort studies, published from April 2011 [24] until June 2022 [25], that reported an association between ideal CVH metrics and the risk of CVD (composite, CHD, and MI), stroke, and CVM (Table 1). There were multiple papers published from the same large population-based studies, like Kuopio Ischemic Heart Disease (KIHD) from Finland [26,27,28], National Health and Nutrition Examination Survey (NHANES) from the USA [29,30,31], and Kailuan from China [20,32,33] (Table 1). The total number of cohort members was 3,240,660, out of which 57.8% were men. Sample sizes ranged from 2520 to 2,728,427 participants (Table 2). There were 3 published articles from the NHANES study that included only males [29,30,31]. Follow-up duration ranged from 3.3 to 26 years (mean 12.0 ± 7.2 years). All studies had NOS scores of 7 or 8 (Table 1).

**Table 1:** Characteristics of the included studies.

Reference, Year	Country	Study Name	Subjects (*n*)	Men (%)	Age (Range or Mean) (y)	Main Outcome	Follow-Up (y)	NOS Score
Fernandez-Lazaro et al., 2022 [25]	Spain	RIVANA	3826	44.1	52.8 ± 12.8	CVD, MI, Stroke, CVM	12.8	8
Itoh et al., 2022 [34]	Japan	JMDC database	2728427	56.2	44.9 ± 11.0	MI, stroke	3.3	8
Isiozor et al., 2021 [26]	Finland	KIHD	2520	100	42–60	Stroke	26	7
Isiozor et al., 2019 [27]	Finland	KIHD	2584	100	40–62	MI	25.2	7
Isiozor et al., 2019 [28]	Finland	KIHD	2607	100	42–60	CVM	25.8	8
Diez-Espino et al., 2019 [21]	Spain	PREDIMED	7447	42.5	67 ± 6.2	MI, stroke, CVM	4.8	8
Ahmad et al., 2019 [29]	USA	NHANES	6766	46.1	59.1 ± 13.3	CVM	14	8
Han et al., 2018 [35]	China	China-PAR	93987	40.2	51.64 ± 11.97	CVD, CHD, stroke, CVM	15	7
Gaye et al., 2017 [36]	N. Ireland, France	PRIME	9312	100	50–59	CHD, stroke	10	7
Gaye et al., 2017 [37]	France	Three-City	7371	36.7	73.82 ± 5.34	CHD, Stroke	8.6	7
Ommerborn et al., 2016 [38]	USA	Jackson Heart	3707	45.1	40–76	CVD	8.3	7
Lachman et al., 2016 [39]	UK	EPIC-Norfolk	10043	44.1	57.0 ± 9.67	CVD, CHD, stroke	10	7
Miao et al., 2015 [20]	China	Kailuan	91598	79.5	51.6 ± 12.4	CVD, MI, stroke	6.8	8
Liu et al., 2014 [32]	China	Kailuan	95429	79.7	51.46 ± 12.46	CVM	4	7
Zhang et al., 2013 [33]	China	Kailuan	91698	79.4	51.93	Stroke	4	7
Kulshreshta et al., 2013 [40]	USA	REGARDS	22915	41.9	65	Stroke	4.9	8
Kim et al., 2013 [41]	S. Korea	Seoul Male Cohort	12538	100	40–59	CVM	19	8
Yang et al., 2012 [30]	USA	NHANES	13312	49	46.8	CVM	14.5	8
Ford et al., 2012 [31]	USA	NHANES	6855	47.7	43 (median)	CVM	5.8	7
Dong et al., 2012 [42]	USA	NOMAS	2981	36.3	69 ± 10	CVD, MI, stroke, CVM	11	8
Artero et al., 2012 [43]	USA	ACLS	11993	75.7	46 ± 9.9	CVM	11.6	8
Folsom et al., 2011 [24]	USA	ARIC	12744	43.8	45–64	CVD	18.7	8

Legend: CVD—cardiovascular disease; MI—myocardial infarction; CVM—cardiovascular mortality; CHD—coronary heart disease; RIVANA—Vascular Risk in Navarra; JMDC—Japan Machine Design Center; KIHD—Kuopio Ischemic Heart Disease; PREDIMED—Prevención con Dieta Mediterránea; NHANES—National Health and Nutrition Examination Survey; PRIME—Prospective Epidemiological Study of Myocardial Infarction; EPIC—European Prospective Investigation into Cancer; REGARDS—Reasons for Geographic And Racial Differences in Stroke; NOMAS—Northern Manhattan Study; ACLS—Aerobics Center Longitudinal Study; ARIC—Atherosclerosis Risk in Communities.

**Table 2:** Summary of analyzed data for each outcome and CVH metrics group.

Events (*n*)	Subjects (*n*, Male %)	Poor (CVH Metrics: 0–2 or Score 0–4)		Intermediate (CVH Metrics: 3–4 or Score 5–9)		Ideal (CVH Metrics: 5–7 or Score 10–14)		Follow-Up (Average, y)
		Subjects	Events	Subjects	Events	Subjects	Events	
**CVD (12069)**	**218786 (57.2)**	18972	3370	110347	6588	89467	2111	11.8 ± 4.1
**CHD (2829)**	120713 (44.9)	13666	993	51972	1537	55075	299	10.9 ± 2.8
**MI (7629)**	2836863 (56.9)	281282	2209	1086848	3754	1468733	1666	10.6 ± 8.0
**Stroke (35190)**	3072125 (57.0)	342422	6539	1196199	17284	1533504	11367	9.8 ± 6.3
**CVM (5500)**	257741 (60.9)	62927	1945	117758	2742	77056	813	12.6 ± 6.4

Legend: CVH—cardiovascular health; CVD—cardiovascular disease; MI—myocardial infarction; CVM—cardiovascular mortality; CHD—coronary heart disease.

Table 2 represents a summary of each cardiovascular outcome and study population, with notably the highest number of events and subjects reported for stroke (the number of stroke events was 35,190, while the subject population was 3.07 million which was followed over the 9.8 ± 6.3 years).

### 4.2. Association between Ideal CVH Metrics and the Risk of Composite CVD

**Figure 2:**
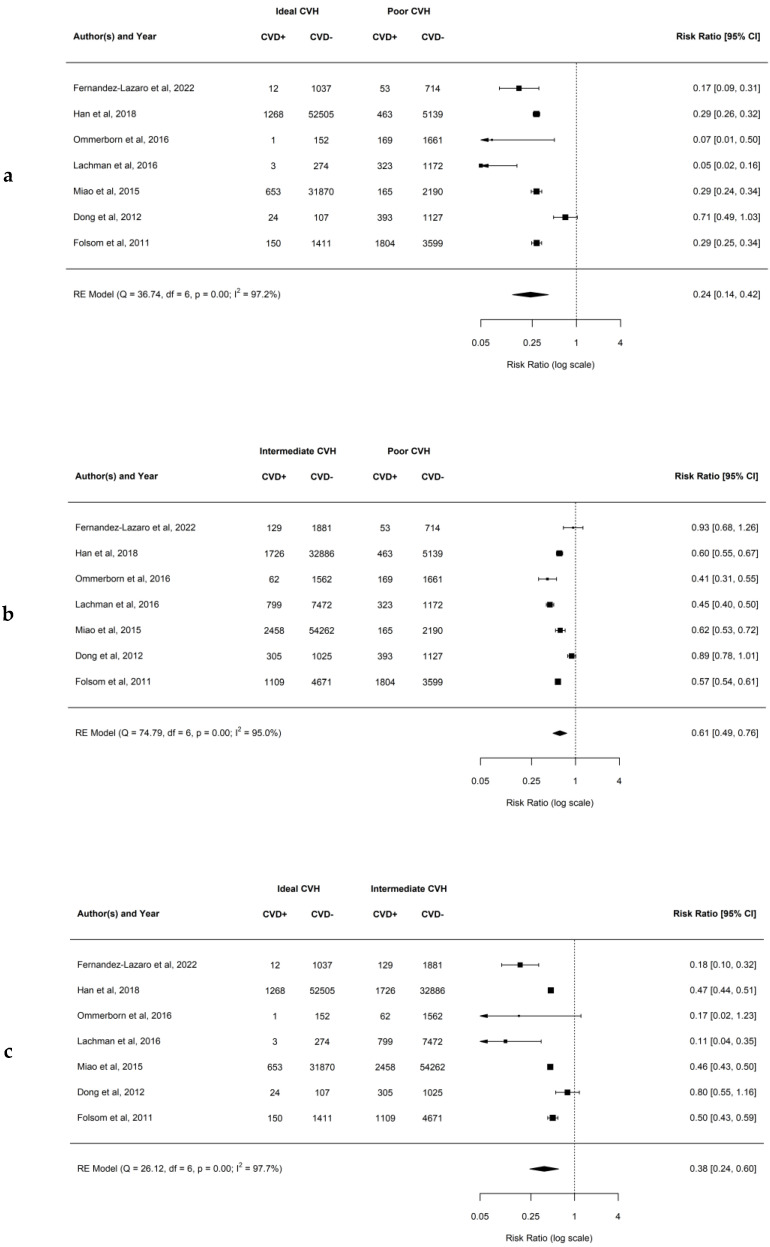
(**a**–**c**) Forest plots showing comparisons of CVH metrics groups for the composite CVD with RR and 95% CI [20,24,25,35,38,39,42].

The results of our analysis demonstrated that having a higher number of ideal CVH metrics decreases the risk of developing composite CVD. When comparing the ideal to poor CVH profile, the observed RR ranged from 0.05 to 0.71, while the estimated average RR based on the RE model was 0.24 (95% CI: 0.14–0.42; *p* < 0.01; *I*^2^ = 97.2%). This demonstrates that there is a 76% lower risk of developing CVD for patients having ideal compared to poor CVH. Similarly, when comparing intermediate to poor CVH groups, the observed RR ranged from 0.41 to 0.93, and the estimated average RR based on the RE model was 0.61 (95% CI: 0.49–0.76; *p* < 0.01; *I*^2^ = 95.0%). This demonstrates that there is a 39% lower risk of developing CVD for patients having intermediate compared to poor CVH. In addition, when comparing ideal to intermediate CVH groups, the observed RR ranged from 0.11 to 0.80, and the estimated average RR based on the RE model was 0.38 (95% CI: 0.24–0.60; *p* < 0.01; *I*^2^ = 97.7%). This demonstrates that there is a 62% lower risk of developing CVD for patients having ideal compared to intermediate CVH. Forest plots showing the observed outcomes and the estimates based on the RE model are shown in Figure 2a–c. Publication bias was not detected, and funnel plots were symmetric (as shown in Supplemental Figures S1–S3 with respective *p* = 0.613, *p* = 0.713, and *p* = 0.391).

### 4.3. Association between Ideal CVH Metrics and the Risk of CHD

**Figure 3:**
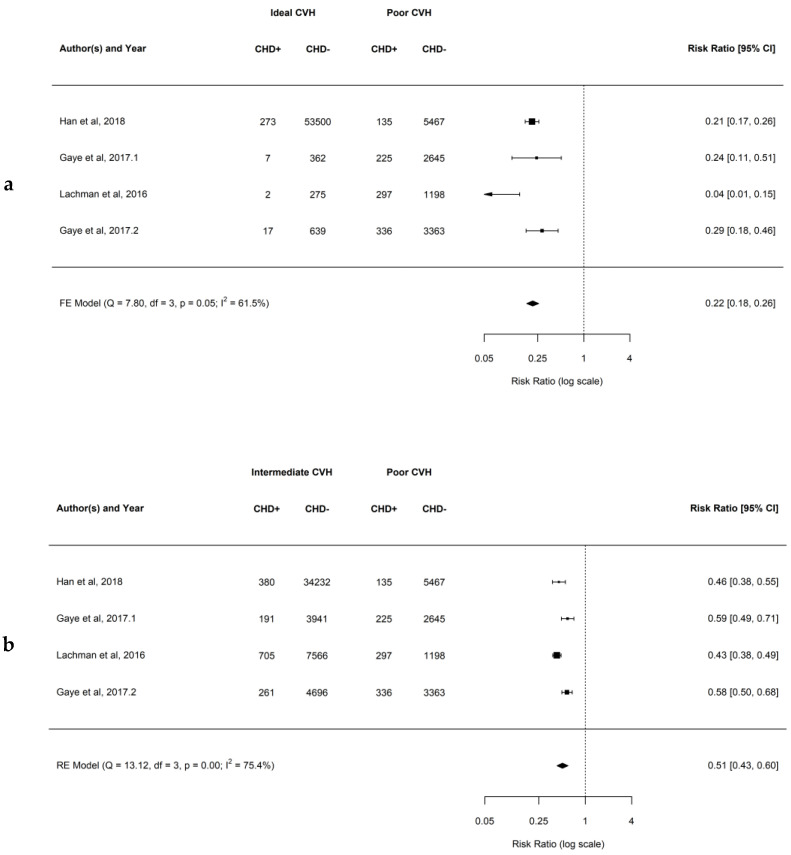

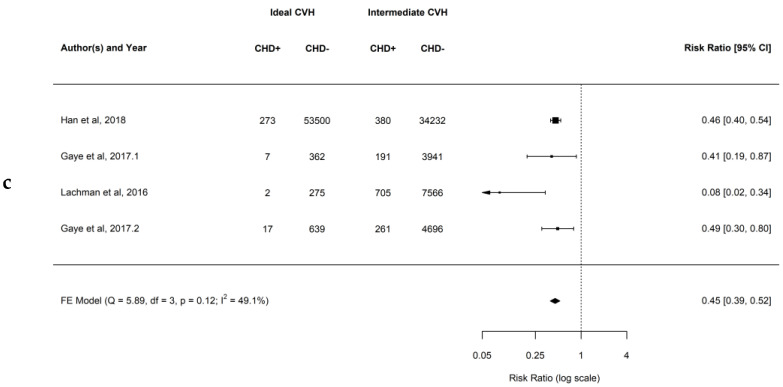
(**a**–**c**) Forest plots showing comparisons of CVH metrics groups for the CHD with RR and 95% CI [35,36,37,39].

Overall, when comparing the individuals with higher numbers of ideal CVH metrics the risk for development of coronary heart disease (CHD) was lower. When comparing ideal to poor CVH groups, the observed RR ranged from 0.04 to 0.29, and the estimated average RR based on the FE model was 0.22 (95% CI: 0.18–0.26; *p* = 0.05; *I*^2^ = 61.5%). This demonstrates that there is a 78% lower risk of developing CHD for patients having ideal compared to poor CVH. Similarly, when comparing intermediate to poor CVH groups, the observed RR ranged from 0.43 to 0.59, with the estimated average RR based on the RE model was 0.51 (95% CI: 0.43–0.60; *p* < 0.01; *I*^2^ = 75.4%). This demonstrates that there is a 49% lower risk of developing CHD for patients having intermediate to poor CVH. In addition, when comparing ideal to intermediate CVH groups, the observed RR ranged from 0.08 to 0.49, with the estimated average RR based on the FE model was 0.45 (95% CI: 0.39–0.52; *p* = 0.12; *I*^2^ = 49.1%). This demonstrates that there is a 55% lower risk of developing CHD for patients having ideal compared to intermediate CVH. Forest plots showing the observed outcomes and the estimates based on the FE and RE models are shown in Figure 3a–c. Publication bias was not detected, and funnel plots were symmetric (as shown in Supplemental Figures S4–S6 with respective *p* = 0.608, *p* = 0.452, and *p* = 0.330).

### 4.4. Association between Ideal CVH Metrics and the Risk of MI

**Figure 4:**
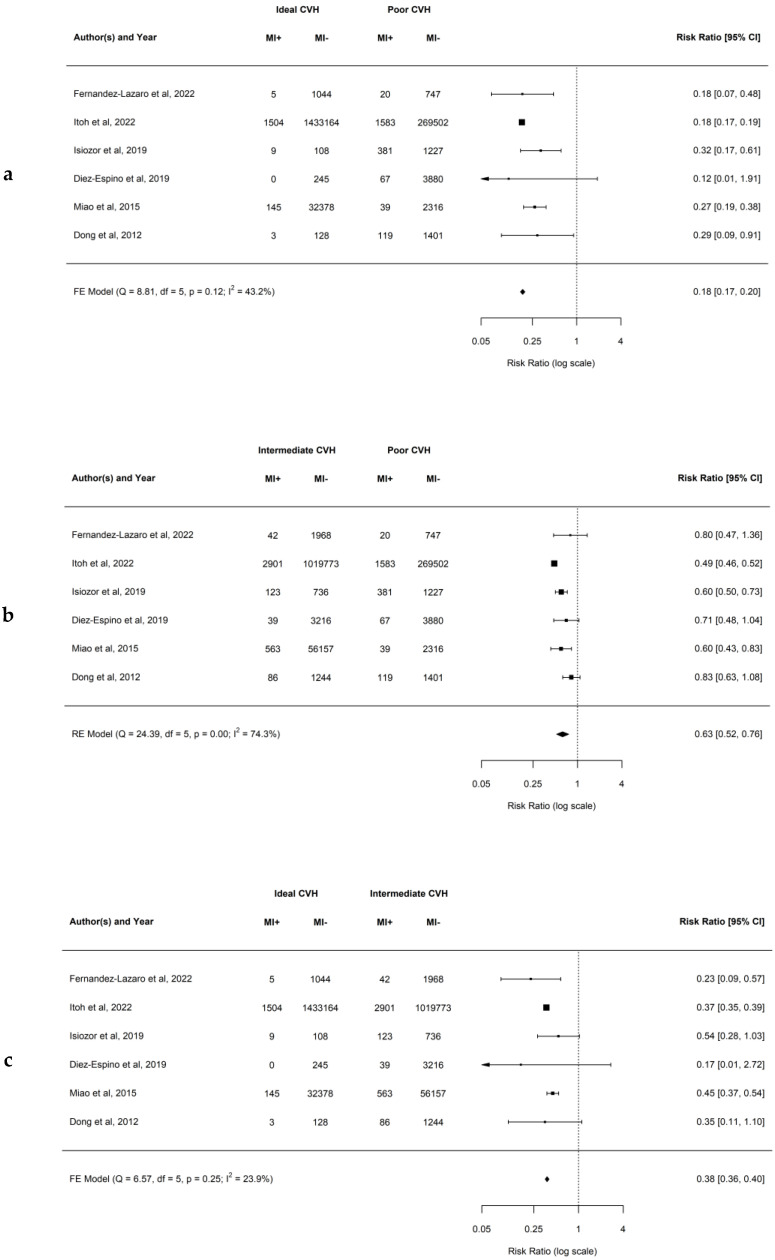
(**a**–**c**) Forest plots showing comparisons of CVH metrics groups for the MI with RR and 95% CI [20,21,25,27,34,42].

The results of our analysis demonstrated that having a higher number of ideal CVH metrics decreases the risk of developing MI. When comparing ideal to poor CVH groups, the observed RR ranged from 0.12 to 0.32, and the estimated average RR based on the FE model was 0.18 (95% CI: 0.17–0.20; *p* = 0.12; *I*^2^ = 43.2%). This demonstrates that there is an 82% lower risk of developing MI for patients having ideal compared to poor CVH. Similarly, when comparing intermediate to poor CVH groups, the observed RR ranged from 0.49 to 0.83, and the estimated average RR based on the RE model was 0.63 (95% CI: 0.52–0.76; *p* < 0.01; *I*^2^ = 74.3%). This demonstrates that there is a 37% lower risk of developing MI for patients having intermediate compared to poor CVH. In addition, when comparing ideal to intermediate CVH groups, the observed RR ranged from 0.17 to 0.54, and the estimated average RR based on the FE model was 0.38 (95% CI: 0.36–0.40; *p* = 0.25; *I*^2^ = 23.9%). This demonstrates that there is a 62% lower risk of developing MI for patients having ideal compared to intermediate CVH. Forest plots showing the observed outcomes and the estimates based on the FE and RE models are shown in Figure 4a–c. Publication bias was not detected for “ideal vs poor” and “ideal vs intermediate”, and funnel plots were symmetric (as shown in Supplemental Figures S7 and S9 with respective *p* = 0.182, and *p* = 0.837), however, the publication bias was detected in “intermediate vs poor” with funnel plot being asymmetric (Supplemental Figure S8 with *p* = 0.015).

The study from Japan Itoh et al. [34] was identified as an influential study in the case of MI when comparing all three CVH groups by providing 96.2% of the subjects. Leave-one-out analysis indicated that the RR excluding Itoh et al. [34] would be 0.27 (95% CI: 0.20–0.36) for ideal vs poor CVH, 0.68 (95% CI: 0.58–0.79) for intermediate vs poor CVH, and 0.44 (95% CI: 0.37–0.52) for ideal vs intermediate CVH.

### 4.5. Association between Ideal CVH Metrics and the Risk of Stroke

**Figure 5:**
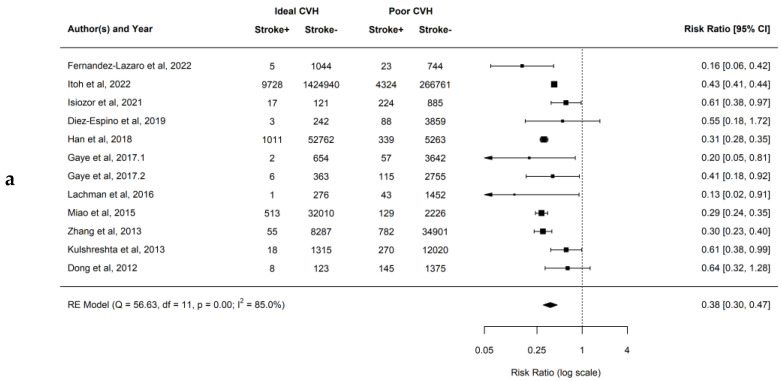

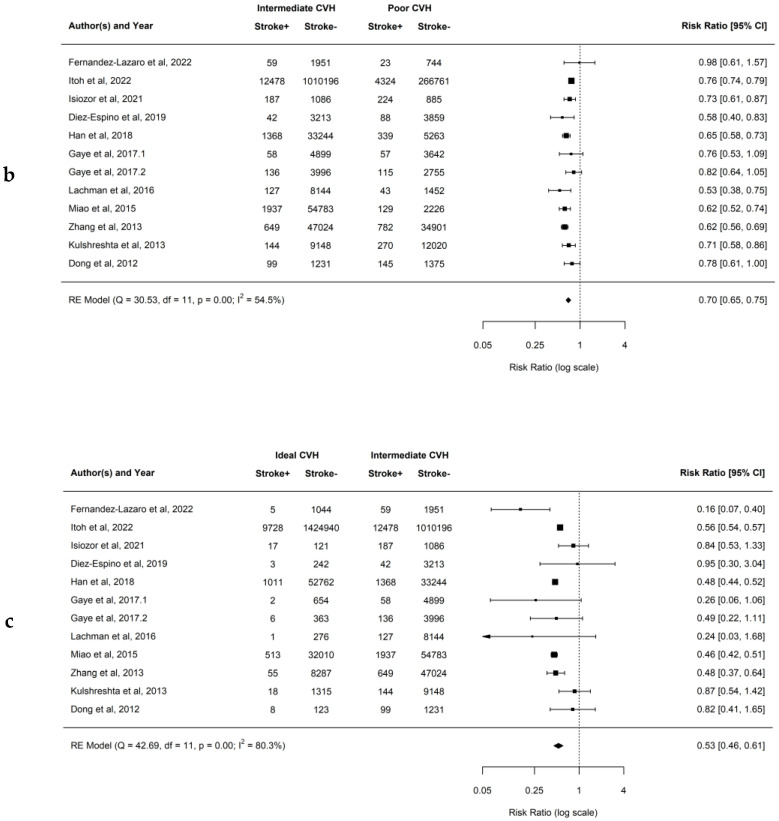
(**a**–**c**) Forest plots showing comparisons of CVH metrics groups for the Stroke with RR and 95% CI [20,21,25,26,33,34,35,36,37,39,40,42].

Overall, when comparing the individuals with a higher number of ideal CVH metrics the risk for the development of stroke was lower. When comparing ideal to poor CVH groups, the observed RR ranged from 0.16 to 0.64, with the estimated average RR based on the RE model was 0.38 (95% CI: 0.30–0.47; *p* < 0.01; *I*^2^ = 85.0%). This demonstrates that there is a 62% lower risk of developing stroke for patients having ideal compared to poor CVH. Similarly, when comparing intermediate to poor CVH groups, the observed RR ranged from 0.53 to 0.98, and the estimated average RR based on the RE model was 0.70 (95% CI: 0.65–0.75; *p* < 0.01; *I*^2^ = 54.5%). This demonstrates that there is a 30% lower risk of developing stroke for patients having intermediate compared to poor CVH. In addition, when comparing ideal to intermediate CVH groups, the observed RR ranged from 0.16 to 0.95, and the estimated average RR based on the RE model was 0.53 (95% CI: 0.46–0.61; *p* < 0.01; *I*^2^ = 80.3%). This demonstrates that there is a 47% lower risk of developing stroke for patients having ideal compared to intermediate CVH. Forest plots showing the observed outcomes and the estimates based on the RE model are shown in Figure 5a–c. Publication bias was not detected, and funnel plots were symmetric (as shown in Supplemental Figures S10–S12 with respective *p* = 0.298, *p* = 0.155, and *p* = 0.498).

Itoh et al. [34] was identified as an influential study in the case of stroke when comparing intermediate vs poor CVH. Leave-one-out analysis indicated that the RR excluding Itoh et al. [34] would be 0.67 (95% CI: 0.63–0.72) compared to 0.70 (95% CI 0.65–0.75) when all studies are included. Although the study by Itoh et al. [34] does influence the effect size in the case of MI and stroke by providing 96.2% and 88.8% of the analyzed subjects, respectively, it does not change the direction of the effect, nor its significance.

### 4.6. Association between Ideal CVH Metrics and the Risk of CVM

**Figure 6:**
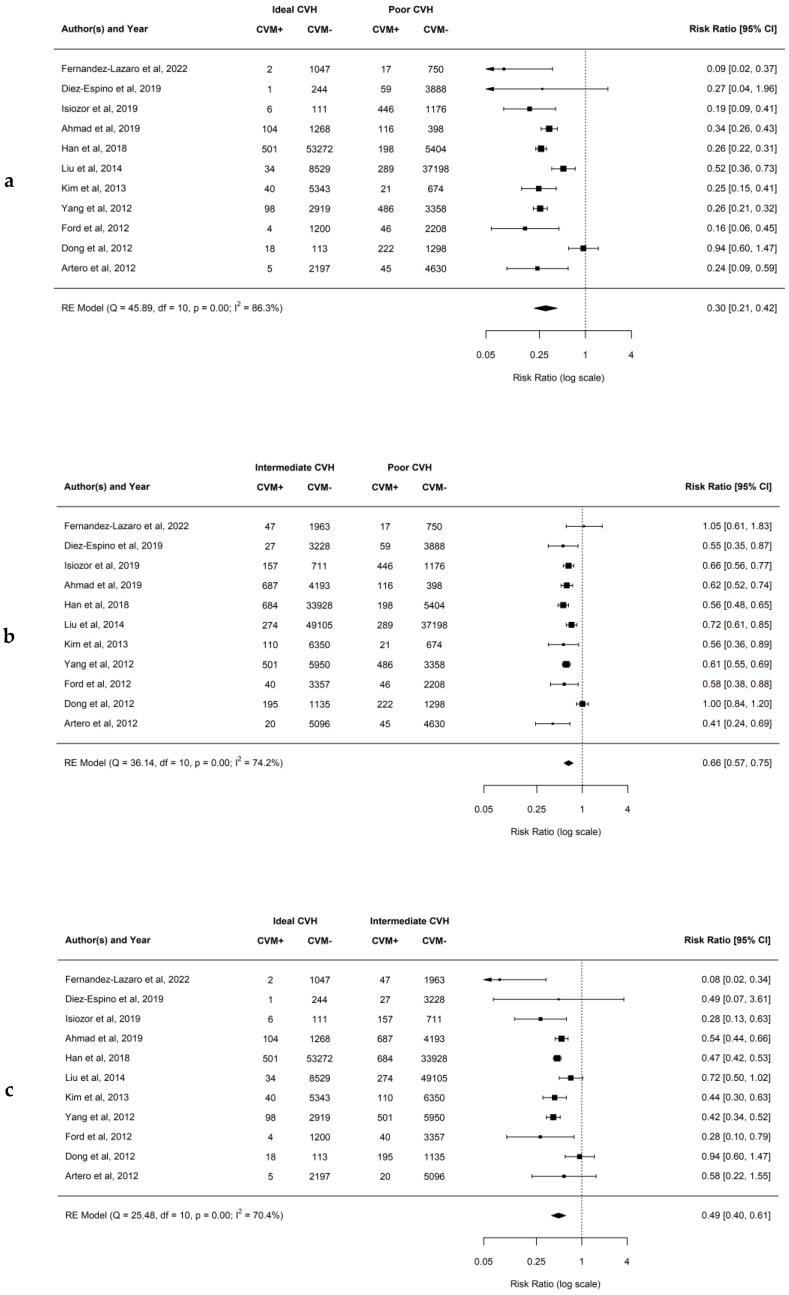
(**a**–**c**) Forest plots showing comparisons of CVH metrics groups for the CVM with RR and 95% CI [21,25,28,29,30,31,32,35,41,42,43].

Results of our analysis demonstrated an inverse relationship showing a reduced risk of CVM with achieving a greater number of ideal CVH metrics. When comparing ideal to poor CVH groups, the observed RR ranged from 0.09 to 0.94, and the estimated average RR based on the RE model was 0.30 (95% CI: 0.21–0.42; *p* < 0.01; *I*^2^ = 86.3%). This demonstrates that there is a 70% lower risk of CVM for patients having ideal compared to poor CVH. Similarly, when comparing intermediate to poor CVH groups, the observed RR ranged from 0.41 to 1.05, while the estimated average RR based on the RE model was 0.66 (95% CI: 0.57–0.75; *p* < 0.01; *I*^2^ = 74.2%). This demonstrates that there is a 34% lower risk of CVM for patients having intermediate compared to poor CVH. In addition, when comparing ideal to intermediate CVH groups, the observed RR ranged from 0.08 to 0.94, and the estimated average RR based on the RE model was 0.49 (95% CI: 0.40–0.61; *p* < 0.01; *I*^2^ = 70.4%). This demonstrates that there is a 51% lower risk of CVM for patients having ideal compared to intermediate CVH. Forest plots showing the observed outcomes and the estimates based on the RE model are shown in Figure 6a–c. Publication bias was not detected, and funnel plots were symmetric (as shown in Supplemental Figures S13–S15 with respective *p* = 0.961, *p* = 0.880, and *p* = 0.765).

## 5. Discussion

Compared to our analysis that included 22 studies, previous systematic reviews and meta-analyses on the topic of CVH [5,6,7,8] analyzed a total of 13 unique studies (ranging from 6 to 12 studies) [20,24,30,31,32,33,36,38,39,40,41,42,43], and lacked some of the important studies [9]. Except for Guo et al. [5], none of the previous meta-analyses and systematic reviews included all of the papers that fulfilled the reported inclusion criteria by the end of the performed search. The rationale for excluding eligible studies was not documented, and the selection criteria (prospective cohort studies in adults, published in the English language, and that analyzed the relationship of CVH and CVD/CVM) did not justify the exclusion of many important studies [9]. Two studies (Fang et al. [5], and Guo et al. [6]) did not examine the effect of meeting the intermediate compared to poor CVH, which is important as intermediate CVH is much more achievable in the general population than ideal CVH. Additionally, these two studies comparing ideal versus poor CVH were concerning with respect to their inconsistent and highly variable CVH group categorization as reported in “Table 1” in both papers [5,6]. Their ideal CVH group was classified as either 4–7, 5–7, or 6–7 CVH metrics; or 10–14 to 12–14 score points. Likewise, their poor CVH group was classified as either 0, 0–1, or 0–2 metrics; or 0–1, 0–2, to 0–4 score points. This variability led authors to compare dissimilar and incomparable categories of CVH. Similarly, in the paper by Aneni et al. [8], the authors compared various levels of CVH with a reference group that was un-uniform and ranged from 0, to 0–1, to 0–2 CVH metrics. This significant heterogeneity engenders substantial concerns about the validity and comparability of study results. Conversely, in our analysis, we strictly categorized data into three CVH metric groups, and if sufficient data were unavailable in the published manuscripts and supplements, we requested additional information from the study authors (from three such papers, we received additional information from only one study [21], excluding the other two studies [22,23]).

The results of our analysis confirm that achieving a higher number of ideal CVH metrics is associated with a lower risk for CVD and CVM. Our findings align with those of previous studies despite their aforementioned methodological limitations [5,6,7,8]. This was derived by comparing the 3 CVH metrics groups, having relative risk reduction with a higher number of achieved CVH metrics. In addition, Aneni et al. [8] reported an inverse linear relationship between CVH metrics and mortality, with an estimated 19% reduction in CVM for each achieved CVH metric. It was also reported in the literature that a longer duration of favorable CVH was associated with decreased cardiovascular-related morbidity and mortality [4].

The low prevalence of ideal CVH is a cause for concern. Amongst our analyzed cohort, only 12.3% qualified as ideal CVH, 39.4% fell into intermediate CVH, and 48.3% were classified as poor CVH. Similar patterns were reported in the published literature of individual studies where ideal CVH was found to be the least common (prevalence of 0.5% to 3.3% reported in the general population [44] but approaching 15% in some subgroups and specific populations [45,46]). Data from NHANES 2011–2016 reported a predominance of poor CVH in the general US population (around 59%), while merely 7.3% of adults had ideal CVH [47]. In another study, about 62% of US adults achieved 3 or fewer CVH metrics [48]. Given that even a single risk factor is associated with an increased lifetime risk of CVD, the present distribution of CVH is troubling [4,49].

Ideal CVH is difficult both to achieve and maintain. As such, it may be more realistic to improve CVH by achieving metrics that can move the population from the poor to the intermediate category. The previous systematic reviews and meta-analyses compared ideal with poor CVH. Our study, however, assessed the comparative benefits of achieving intermediate over poor CVH, which we believe to be a more attainable goal. We went one step further by assessing the risk reduction of achieving the ideal CVH in comparison to intermediate CVH, and the implications resulting from that additional improvement. The only other study that studied intermediate CVH was Ramírez-Vélez et al. [7]; however, they did not analyze ideal versus intermediate CVH, nor did they assess the effect of various CVH levels on CVM. For the composite CVD, CHD, MI, and stroke, our study reported similar results as Ramírez-Vélez et al. [7], despite our analysis of 8 additional studies (our analysis comprised a total of 22 studies; 15 after excluding studies reporting CVM, while Ramírez-Vélez et al. had 12 studies; 7 after excluding studies reporting heart failure and venous thromboembolism, which they analyzed). In both our study and that of Ramírez-Vélez et al. [7], there was a significantly lower risk when higher levels of CVH were compared to lower CVH. When comparing ideal to poor CVH, our results demonstrated that there was a lower risk of developing composite CVD, CHD, MI, and stroke of 76%, 78%, 82%, and 62%, respectively. Similarly, Ramírez-Vélez et al. [7] reported a lower risk for developing composite CVD, CHD, MI, and stroke of 77%, 79%, 76%, and 67%, respectively. When comparing intermediate to poor CVH, our results demonstrated that there was a lower risk of developing composite CVD, CHD, MI, and stroke of 39%, 49%, 37%, and 30%, respectively. Likewise, Ramírez-Vélez et al. [7] reported a lower risk for developing composite CVD, CHD, MI, and stroke of 55%, 44%, 46%, and 42% respectively. These results demonstrate that while ideal CVH confers the greatest risk reduction for CVD and stroke, achieving the more attainable goal of intermediate CVH (defined as 3–4 metrics or a score of 5–9) still offers substantial protective benefit. Due to the prevalence of poor CVH amongst the general population, Ramírez-Vélez et al. [7] point out that a realistic short-term goal should be the promotion of meeting 3 to 4 CVH metrics in order to achieve a positive outcome. The findings from our study fully support this conclusion and recommendation.

Although the beneficial effects of ideal CVH have been supported by increasing scientific evidence, the precise relationship is still not well measured. Furthermore, strong evidence of individual CVH metrics in relation to CVD, stroke, and mortality is lacking. Preliminary data on socioeconomic, gender, and racial inequalities report the unsatisfactory prevalence of ideal CVH metrics, with significant room for improvement [44]. There are many identified social determinants of health that influence an individual’s psychological health and well-being, which in turn may positively or negatively affect CVH through the continuous interplay of mind-heart-body connections [45]. While a healthier lifestyle from a young age is a successful strategy for higher CVH later in life, the ability to choose and practice healthier lifestyles across the lifespan is strongly influenced by psychosocial health factors [45,50,51,52,53,54,55]. Despite CVD and CVM being improved in the US over the past decade, concerning disparities persist regarding risk factors, health behaviors, and CVM based on ethnicity, race, geography, and income [3]. Similar disparities are present globally, further efforts set by AHA (“2030 Impact Goals”) and WHO are intended to improve health equity and address a broader range of factors that contribute to CVH [3].

## 6. Strengths and Limitations of the Study

The principal strengths of our study are its large study cohort (*n* = 3,240,660), that it has studied various CVD outcomes (including CVM) across all CVH metrics groups, and its comparison of each CVH metric group to the others. Our data interpretation, however, has several limitations that must be considered. In some cases, there was significant heterogeneity between analyzed studies, which was addressed by using the RE model. Measurements of physical activity and diet are not standardized amongst other CVH metrics, and there may have been different interpretations of ideal physical activity levels and diet. We acknowledge there is an overlap of articles published from the same studies and examined patient cohorts, however, a “leave-one-out” analysis showed no significant difference when any one study is excluded from statistical analysis. Not all eligible studies were included due to a lack of data for at least one CVH metrics group and the unavailability of authors to provide the requested information [22,23].

## 7. Conclusions

The results of our study clearly demonstrate that higher adherence to ideal CVH standards yields a significantly lower risk of CVD and CVM. While achieving ideal CVH metrics is associated with the lowest risk, it is imperative to recognize that achieving intermediate CVH metrics will also offer a strong protective effect. Given that the majority of the population has poor CVH, there exists tremendous potential to improve outcomes worldwide. We advocate for sending a strong public health message that even modest improvements in CVH are associated with substantial cardiovascular morbidity and mortality benefits. To that end, we should collectively promote healthier lifestyles and behaviors.

## Data Availability

Not applicable.

## Supplementary Materials

The following supporting information can be downloaded at: https://www.mdpi.com/article/10.3390/jcm12134417/s1. Supplement Table S1. Definition of the cardiovascular health metrics and scores according to the American Heart Association. Supplement Figures S1–S3. Funnel plot publication bias for different categories of cardiovascular health and composite cardiovascular disease risk. Supplement Figures S4–S6. Funnel plot publication bias for different categories of cardiovascular health and coronary heart disease risk. Supplement Figures S7–S9. Funnel plot publication bias for different categories of cardiovascular health and myocardial infarction risk. Supplement Figures S10–S12. Funnel plot publication bias for different categories of cardiovascular health and stroke risk. Supplement Figures S13–S15. Funnel plot publication bias for different categories of cardiovascular health and cardiovascular mortality.
