# Supplementary material for: Ideal Cardiovascular Health and Risk of Cardiovascular Events or Mortality: A Systematic Review and Meta-Analysis of Prospective Studies
Source: J Clin Med. 2023 Jun 30;12(13):4417. doi: 10.3390/jcm12134417 (PMC10342759; doi:10.3390/jcm12134417)
Supplement: Supplementary file 1 [file jcm-12-04417-s001.zip › jcm-2470155-supplementary.docx]

**SUPPLEMENT**

**Search strings:**

The search string used for the review in PubMed was: ((cardiovascular health metrics[Title]) OR (ideal cardiovascular health[Title]) OR (Life’s Simple 7[Title])) AND ((mortality[Title]) OR (death[MESH]) OR (cardiovascular diseases[MESH]) OR (stroke[MESH]) OR (cerebrovascular disease[Title]) OR (coronary heart disease[Title])) with filters: English, from 2010/1/1 - 2022/6/30;

The search string used for the review in the Scopus database was: (TITLE(cardiovascular AND health AND metrics) OR TITLE(ideal AND cardiovascular AND health) OR TITLE(life’s AND simple 7) AND TITLE(mortality) OR ALL(death) OR ALL(cardiovascular AND diseases) OR ALL(stroke) OR TITLE(cerebrovascular AND diseases) OR TITLE(coronary AND heart AND disease)) AND PUBYEAR > 2009 AND (LIMIT-TO (LANGUAGE, "English")).

**Supplement Table 1**. Definition of the cardiovascular health metrics and scores according to the American Heart Association.

| **Cardiovascular health metric** | **Level / Categories points** | | |
| --- | --- | --- | --- |
|  | **Poor / 0 points** | **Intermediate / 1 point** | **Ideal / 2 points** |
| **Smoking** | Current | Former, quit ≤12 months | Never or quit >12 months |
| **Body mass index** | ≥30 kg/m^2^ | 25-29.99 kg/m^2^ | <25 kg/m^2^ |
| **Physical activity** | No exercise | 1-149 min of moderate exercise  or 1-74 min of vigorous exercise/week | 150+ min of moderate exercise or 75+ min of vigorous exercise/week |
| **Diet** | 0-1 components of a healthy diet | 2-3 components of a healthy diet | 4-5 components of a healthy diet |
| **Total cholesterol** | ≥240 mg/dL | 200–239 mg/dL or treated to goal | <200 mg/dL, untreated |
| **Blood pressure** | SBP ≥140 or DBP ≥90 mmHg | SBP 120–139 mmHg or DBP 80–89 mmHg or treated to goal | SBP/DBP <120/80 mmHg, untreated |
| **Fasting glucose** | ≥126 mg/dL | 100–125 mg/dL or treated to goal | <100 mg/dL, untreated |

Legend: SBP - systolic blood pressure; DBP - diastolic blood pressure.

**Supplement Figures 1-3. Funnel plot publication bias for different categories of cardiovascular health and composite cardiovascular disease risk.**


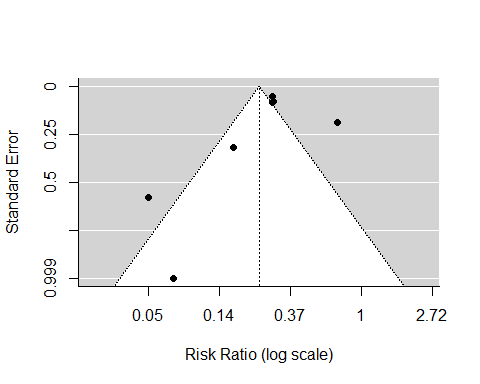


Figure 1. Funnel plot publication bias for ideal vs poor CVH for the composite cardiovascular disease. Publication bias was not detected, and the funnel plot is symmetric (p=0.613).


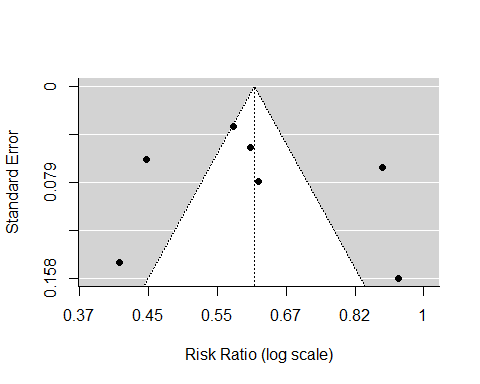


Figure 2. Funnel plot publication bias for intermediate vs poor CVH for the composite cardiovascular disease. Publication bias was not detected, and the funnel plot is symmetric (p=0.713).


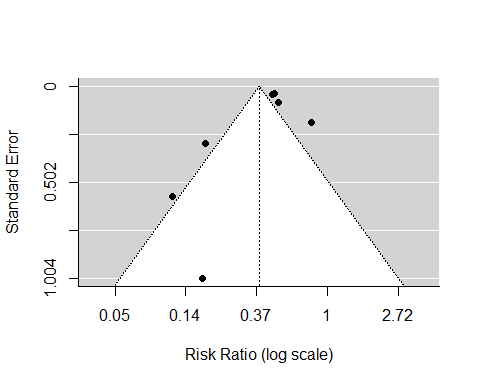


Figure 3. Funnel plot publication bias for ideal vs intermediate CVH for the composite cardiovascular disease. Publication bias was not detected, and the funnel plot is symmetric (p=0.391).

**Supplement Figures 4-6. Funnel plot publication bias for different categories of cardiovascular health and coronary heart disease risk.**


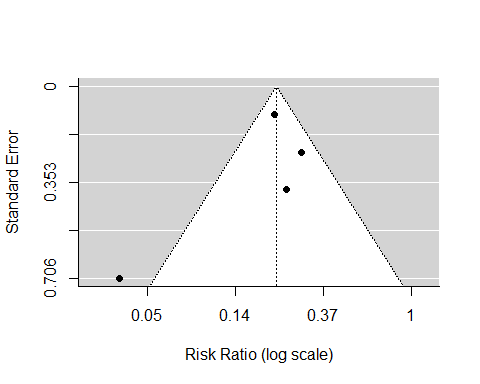


Figure 4. Funnel plot publication bias for ideal vs poor CVH for coronary heart disease. Publication bias was not detected, and the funnel plot is symmetric (p=0.608).


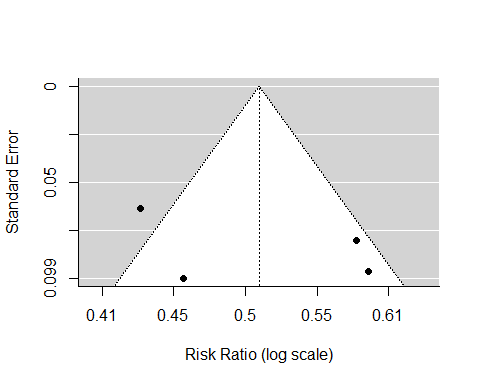


Figure 5. Funnel plot publication bias for intermediate vs poor CVH for coronary heart disease. Publication bias was not detected, and the funnel plot is symmetric (p=0.452).


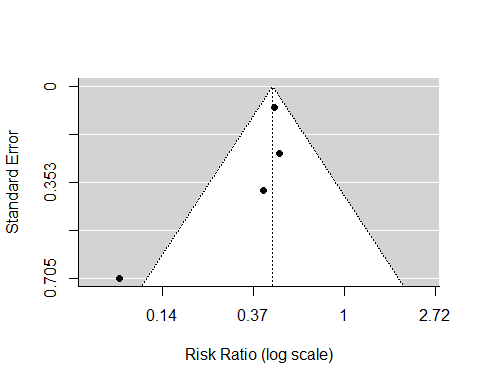


Figure 6. Funnel plot publication bias for ideal vs intermediate CVH for coronary heart disease. Publication bias was not detected, and the funnel plot is symmetric (p=0.330).

**Supplement Figures 7-9. Funnel plot publication bias for different categories of cardiovascular health and myocardial infarction risk.**


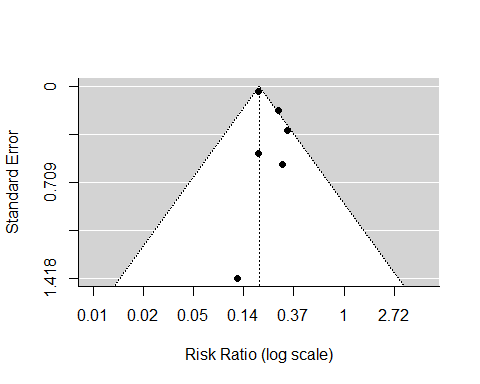


Figure 7. Funnel plot publication bias for ideal vs poor CVH for myocardial infarction. Publication bias was not detected, and the funnel plot is symmetric (p=0.182).


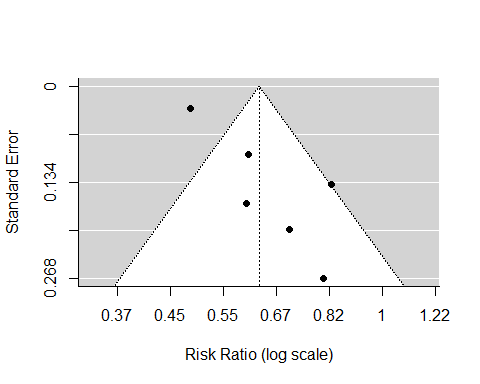


Figure 8. Funnel plot publication bias for intermediate vs poor CVH for myocardial infarction. Publication bias is detected, and the funnel plot is asymmetric (p=0.015).


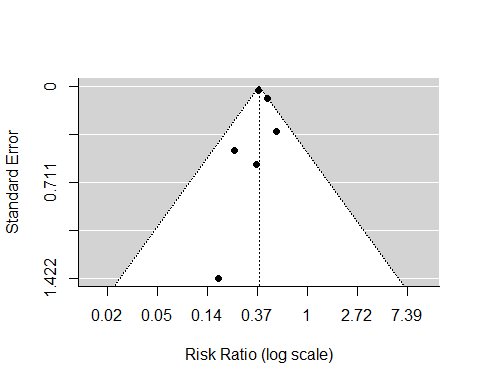


Figure 9. Funnel plot publication bias for ideal vs intermediate CVH for myocardial infarction. Publication bias was not detected, and the funnel plot is symmetric (p=0.837).

**Supplement Figures 10-12. Funnel plot publication bias for different categories of cardiovascular health and stroke risk.**


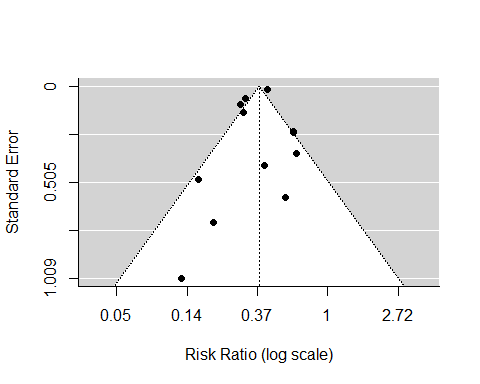


Figure 10. Funnel plot publication bias for ideal vs poor CVH for stroke. Publication bias was not detected, and the funnel plot is symmetric (p=0.298).


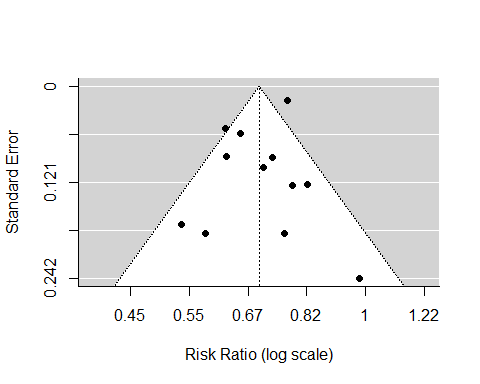


Figure 11. Funnel plot publication bias for intermediate vs poor CVH for stroke. publication bias is not detected, and the funnel plot is symmetric (p=0.155).


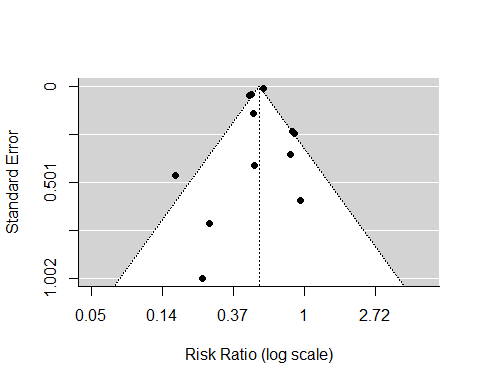


Figure 12. Funnel plot publication bias for ideal vs intermediate CVH for stroke. Publication bias is not detected, and the funnel plot is symmetric (p=0.498).

**Supplement Figures 13-15. Funnel plot publication bias for different categories of cardiovascular health and cardiovascular mortality.**


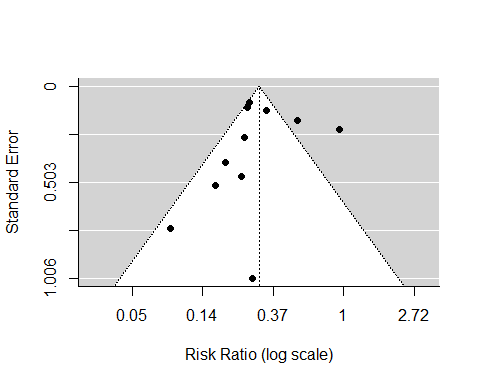


Figure 13. Funnel plot publication bias for ideal vs poor CVH for cardiovascular mortality. Publication bias is not detected, and the funnel plot is symmetric (p=0.961).


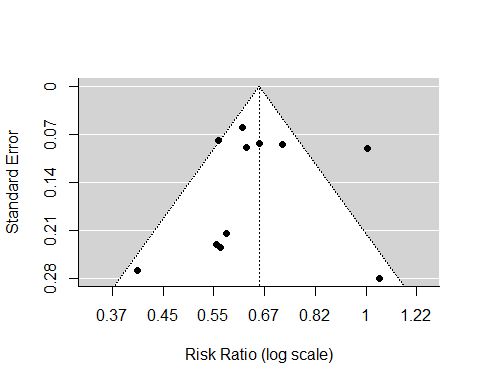


Figure 14. Funnel plot publication bias for intermediate vs poor CVH for cardiovascular mortality. Publication bias is not detected, and the funnel plot is symmetric (p=0.880).


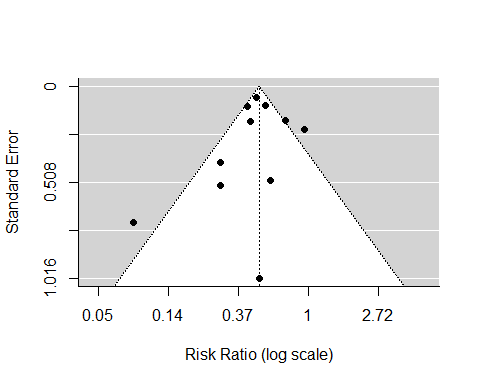


Figure 15. Funnel plot publication bias for ideal vs intermediate CVH for cardiovascular mortality. Publication bias is not detected, and the funnel plot is symmetric (p=0.765).
